# Influence of grain size and composition, topology and excess free volume on the deformation behavior of Cu–Zr nanoglasses

**DOI:** 10.3762/bjnano.6.56

**Published:** 2015-02-24

**Authors:** Daniel Şopu, Karsten Albe

**Affiliations:** 1 Institut für Materialwissenschaft, Technische Universität Darmstadt, Petersenstr. 32, D-64287 Darmstadt, Germany; 2 Currently at: IFW Dresden, Helmholtzstr. 20, 01069 Dresden, Germany

**Keywords:** enhanced plasticity, metallic glasses, nanoglasses, shear bands

## Abstract

The influence of grain size and composition on the mechanical properties of Cu–Zr nanoglasses (NGs) is investigated by molecular dynamics simulations using two model glasses of different alloy composition, namely Cu_64_Zr_36_ (Cu-rich) and Cu_36_Zr_64_ (Zr-rich). When the grain size is increased, or the fraction of interfaces in these NGs is decreased, we find a transition from a homogeneous to an inhomogeneous plastic deformation, because the softer interfaces are promoting the formation shear transformation zones. In case of the Cu-rich system, shear localization at the interfaces is most pronounced, since both the topological order and free volume content of the interfaces are very different from the bulk phase. After thermal treatment the redistribution of free volume leads to a more homogenous deformation behavior. The deformation behavior of the softer Zr-rich nanoglass, in contrast, is only weakly affected by the presence of glass–glass interfaces, since the interfaces don’t show topological disorder. Our results provide clear evidence that the mechanical properties of metallic NGs can be systematically tuned by controlling the size and the chemical composition of the glassy nanograins.

## Introduction

A nanoglass (NG) is a nanostructured material produced via cold compaction of glassy nanoparticles [1]. It consists of glassy grains separated by glass–glass interfaces. Indirect experimental evidence for the existence of interfaces in NGs have been provided by Gleiter et al. [2–7]. The recent work of Chen et al. [8], supports Gleiter’s results on the structural model of a NG. The microstructure of a metallic nanoglass consisting of glassy grains and glass–glass interfaces has been experimentally revealed by electron microscopy, small-angle X-ray scattering and positron annihilation spectroscopy [9], while molecular dynamics studies showed that glass–glass interfaces exhibit an excess free volume and a modified local order [10–11]. If plastically deformed, the soft glass in the interfaces promotes shear band nucleation similar to the effect of residual shear bands in pre-deformed metallic glasses [11]. Consequently, the NG exhibits a more homogeneous plastic deformation carried by a pattern of multiple shear bands [12] as compared to the bulk metallic glass (BMG), where plastic deformation is well localized in a few dominant shear bands. The influence of interfaces on the deformation behavior of nanoglasses has been shown both in computer simulation [11,13–14] and experiment [9,15], where also an enhanced plasticity under compression was observed indicating that not critical shear bands occur. Recent experiments on sputtered nanograined Au-based glasses also showed high hardness and a low elastic modulus as compared to their bulk counterparts [8].

In light of these interesting results, further studies on the mechanical properties of this new type of material seem to be mandatory. Critical questions are, if and how mechanical properties of NGs change by varying the grain size and grain composition. Moreover, it is unclear how thermal treatment or mechanical pre-deformation affect the plasticity of NGs.

Recently, Adibi et al. [14,16] showed for 2D perodic systems that the plasticity of Cu–Zr NGs of different composition will improve with decreasing grain sizes. In these simulations, however, free surfaces were present that promote the activation of shear transformation zones [17]. Thus, these results are representative for samples on the nanoscale, where surfaces and glass–glass interfaces are competing heterogeneities, but don’t give necessarily a clear picture on the situation in bulk NGs.

In this work, we study the influence of grain size and chemical composition on mechanical properties of Cu–Zr NGs by means of molecular dynamics simulations. First, we investigate whether by varying the grain size the plastic deformation of NGs changes. Second, we investigate if the plastic behavior of NGs changes with glass composition. Finally, the impact of the thermal relaxation of the glass–glass interface is studied. For this, two model glasses of different alloy composition are used, namely Cu_64_Zr_36_ (Cu-rich) and Cu_36_Zr_64_ (Zr-rich). All simulations are carried out in 3D periodic arrangements and thus surface effects are deliberately excluded.

## Methods

Molecular dynamics simulations are carried out with the program package LAMMPS [18]. The modified Finnis–Sinclair type potential for Cu–Zr proposed by Mendelev et al. [19] was applied for describing the atomic interactions. For all simulations a constant integration time step of 2 fs was used. In the first step, two homogeneous amorphous alloys consisting of 8000 atoms were generated by quenching from the melt. Initially, the melts were relaxed at 2000 K for 2 ns to ensure chemical homogeneity. The Cu_64_Zr_36_ and Cu_36_Zr_64_ glassy alloys have been cooled to 50 K with a cooling rate of 0.01 K/ps.

The Cu-centered full icosahedral (FI) short range order (SRO) and free volume content was analyzed using the Voronoi tessellation method [20]. We have focused the characterization of topological SRO on the population of Cu-centered FI [0,0,12,0] because this polyhedron is known to be the key structural motif in amorphous Cu–Zr alloy, characterized by a high packing density [21] and high shear resistance [22].

For modeling the NG microstructure, a 2D array of columnar grains with a hexagonal cross section is used. The grains with diameters of 4 nm, 10 nm and 16 nm have been cut from the glassy bulk structure, respectively. The nanoglass was then generated by compacting the columnar grains under external hydrostatic pressure of 3 GPa at 50 K. An overview of the NGs is presented in Table 1.

**Table 1:** Number of glassy grains, average grain diamater and total number of atoms used for the construction of NGs.

Composition	Grains	<*d>* (nm)	N_atoms_

Cu_64_Zr_36_	81	4	≈8.0 · 10^5^
	18	10	≈5.5 · 10^5^
	18	16	≈2.0 · 10^6^
	BMG		≈1.3 · 10^6^
Cu_36_Zr_64_	18	10	≈1.0 · 10^6^
	BMG		≈1.3 · 10^6^

We simulated the deformation of these NGs and compared to the case of homogeneous BMGs by deforming in uniaxial tension parallel to the *z*-direction with a constant strain rate of 4 · 10^7^ s^−1^ at *T* = 50 K. 3D periodic boundary conditions were applied to exclude surface effects. The pressure in *x* and *y* direction was kept at 0 kbar allowing for lateral contraction. The atomic scale deformation mechanisms were analyzed by visualizing the local atomic shear strain [23], calculated with the OVITO analysis and visualization software [24]. A quantitative interpretation of strain localization has been realized by using the strain localization parameter defined by Cheng et al. [25],


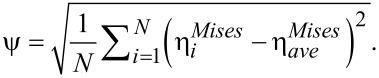


A larger ψ value indicates larger fluctuations in the atomic strain and a more localized deformation mode.

## Results

### Grain size effects

**Figure 1:**
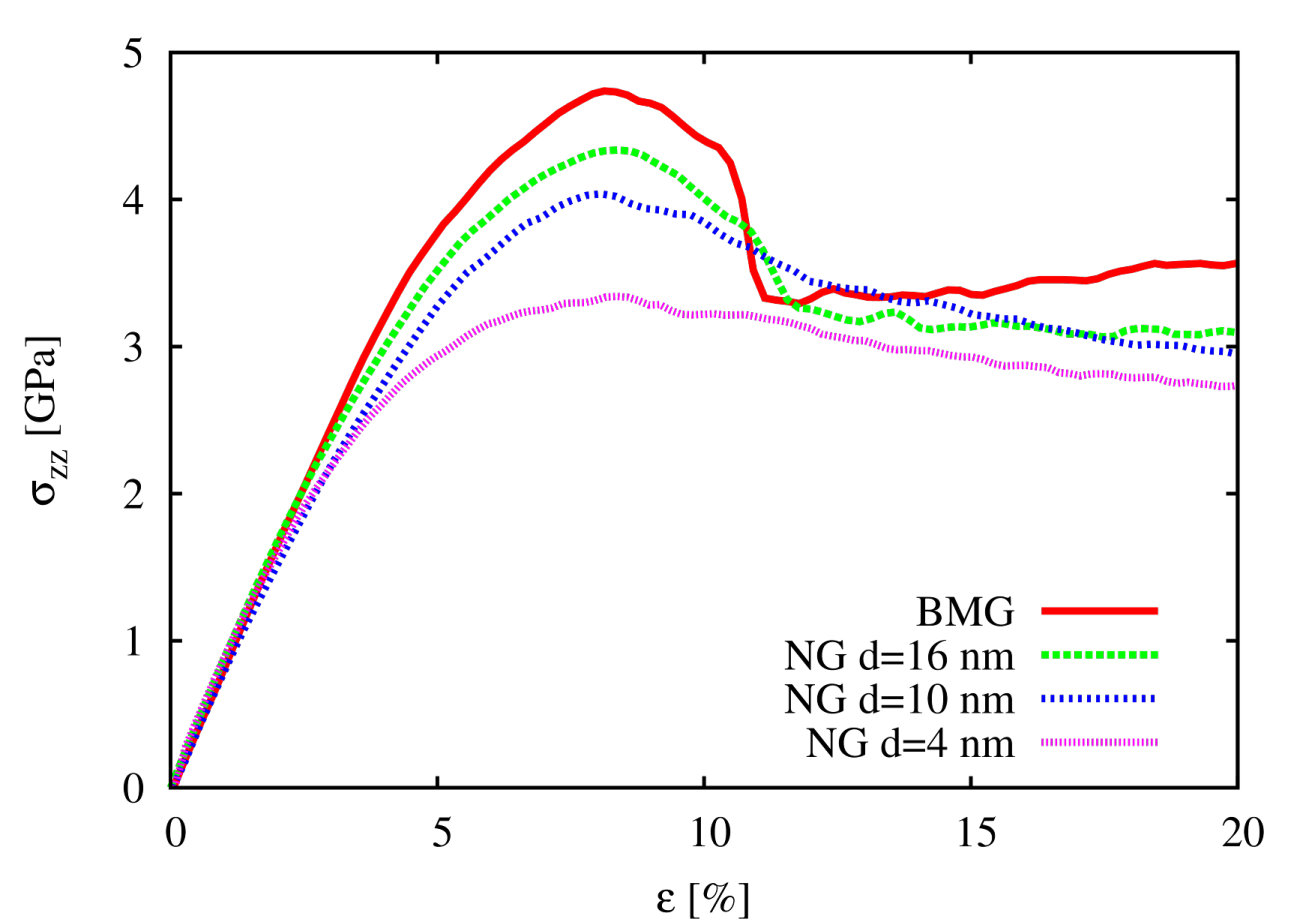
Tensile stress–strain curves for a BMG in comparison to three NGs with grain sizes of 4 nm, 10 nm and 16 nm, respectively, at a constant strain rate of 4 · 10^7^ s^−1^.

For studying the effect of grain size on the mechanical properties, we simulated the deformation of Cu-rich glasses with grain sizes of <*d*> = 4, 10 and 16 nm (see Table 1). All NGs and a BMG (≈1.3 · 10^6^ atoms) were deformed in tension following the procedure described above. The stress–strain curves of all three NGs and the BMG under tensile deformation are plotted in Figure 1. Up to a strain of about 2%, all four curves show a similar slope and, thus, the fraction of interfaces does not have a strong influence on the elastic deformation. In contrast, the yield stress and maximum stress decrease considerably with decreasing grain size or increasing the glass–glass interface density. In the past, we have shown that these planar interfaces have a lower fraction of densely packed FI clusters [11]. As the FI clusters have a high shear resistance [21–22], a decrease in the total amount of FI clusters by increasing the fraction of interfaces in the NG can explain the decrease in yield stress. Therefore, an increased number of interfaces results in a softer NG. In addition, an increase of the volume fraction of interfaces in the NG results in an improved plastic behavior. As shown in our previous work [12], a large number of interfaces causes a more homogeneous deformation of the NG in a highly organized pattern of multiple shear bands, in contrast to the BMG which exhibits localized deformation in one dominant shear band.

**Figure 2:**
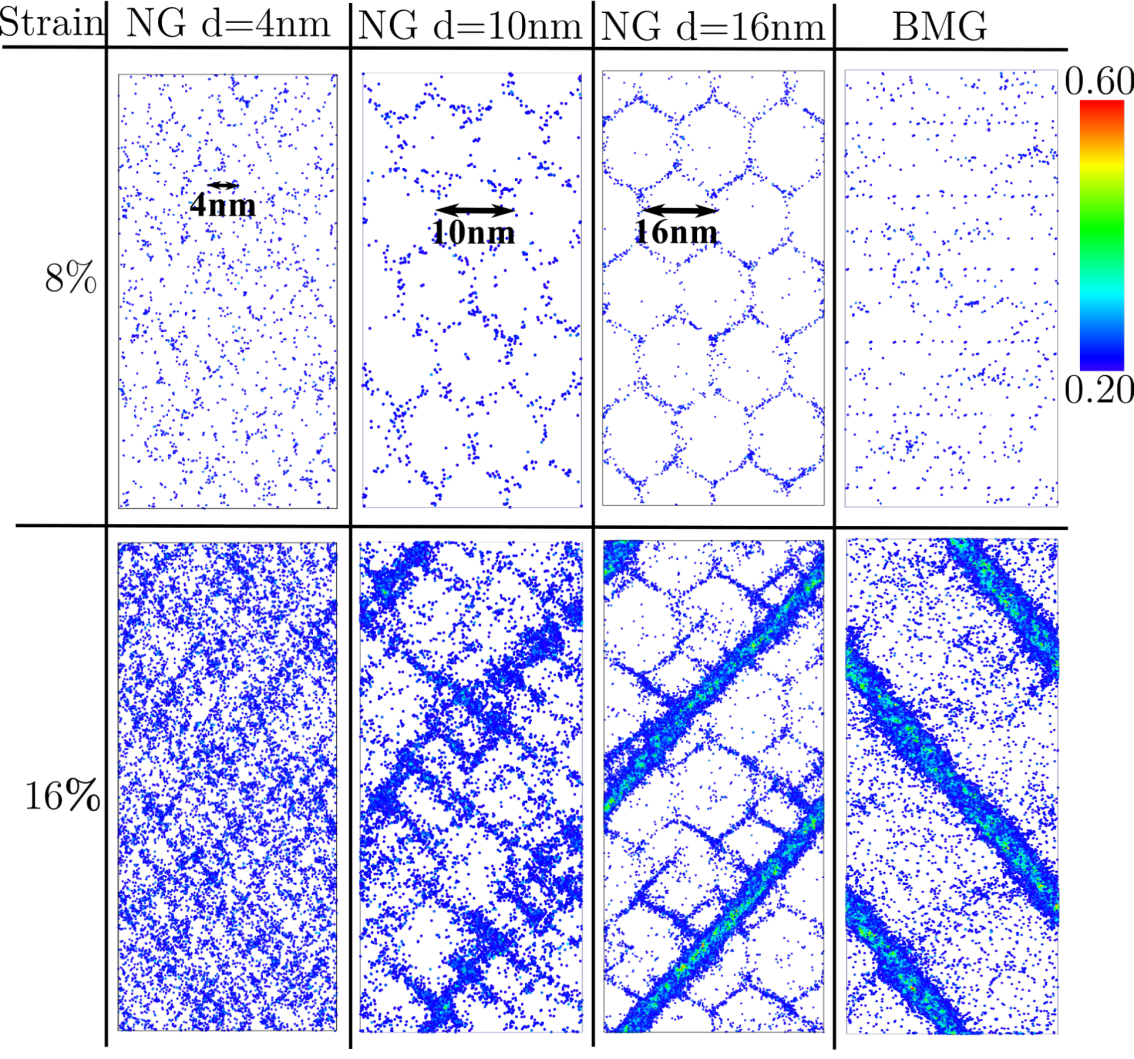
Local atomic shear strain for Cu_64_Zr_36_ BMG and NGs with grain sizes of 4 nm, 10 nm and 16 nm, respectively.

In order to study how the deformation mechanism of NGs changes by varying the glassy grain size the local atomic shear strain was calculated for each of these NGs and BMG (Figure 2). Up to a strain of 8% in all three NGs, the shear transformation zones (STZs) are mostly activated in the soft interface regions. Although the shear band nucleation process is similar in all three NGs, the shear band propagation in case of the NG with the largest grain size strongly differs from to the other two NGs. It can be seen in Figure 2 that the plastic deformation is more localized at a strain of 16% in case of the NG with a grain size of 16 nm. This can be explained by the lower fraction of soft interfaces with respect to the NG volume. Up to a strain of 8%, the elastic energy is released locally in embryonic shear bands formed by precipitation of the STZs along the interfaces (see Figure 2).

**Figure 3:**
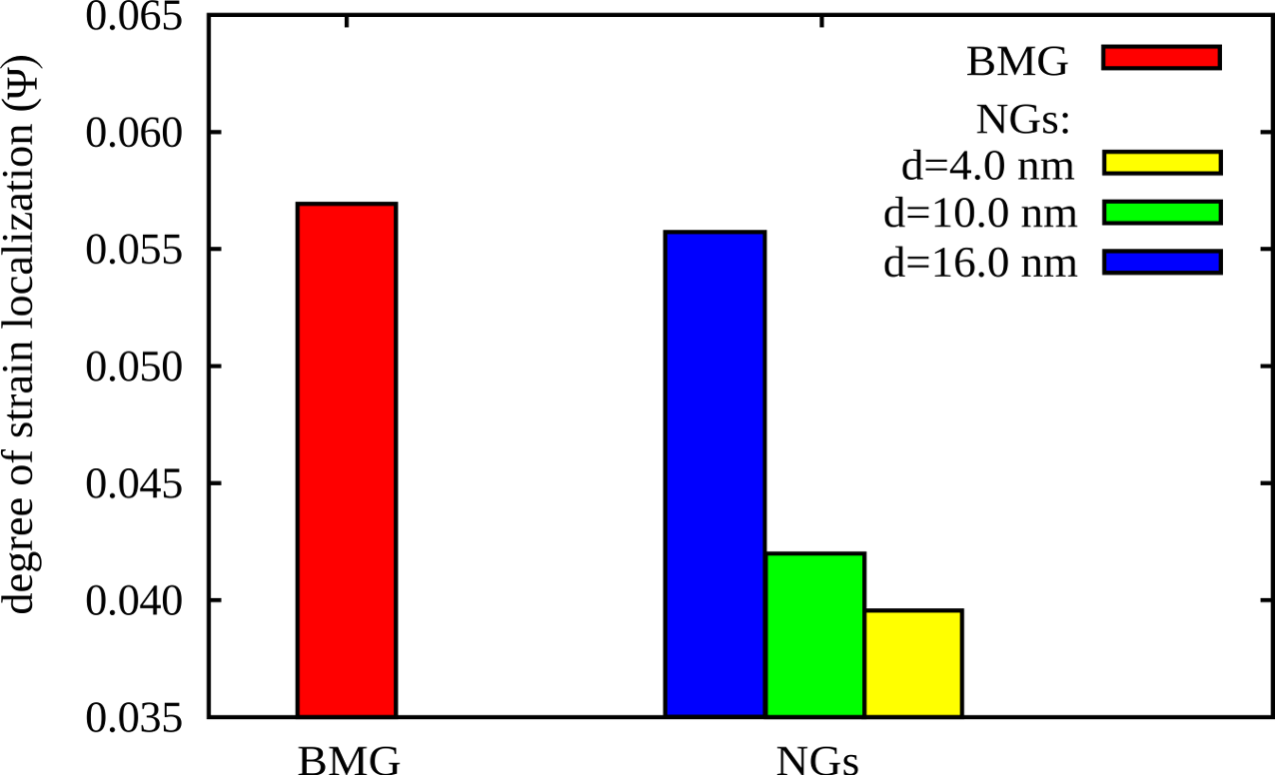
The ψ values for a BMG in comparison to three NGs with grain sizes of 4 nm, 10 nm and 16 nm, respectively, after plastic deformation to an overall strain of 16%.

After a strain of 8% the local energy released is sufficient to accelerate one of these embryonic shear bands, so that it goes critical. The propagation of one single shear band leads to a stress drop, which is not observed for the other two NGs with smaller grains, as it can be seen in Figure 1. Nevertheless, this stress drop is not as pronounced as in the case of the BMG. This means that even for a NG constructed with bigger glassy grains, the deformation deviates from the well localized deformation found in the BMG. Together with a dominant shear band, many secondary shear bands mediate the plastic deformation of the NG (see Figure 2). This observation is supported by the calculated strain localization parameter ψ. The ψ values for all three NGs and BMG at a strain of 16% are plotted in Figure 3. It can be seen that the ψ value of the NG with a grain size of 16 nm is higher than for the other two NGs, but still lower than for the BMG. For the other two NGs with smaller glassy grains the ψ values are much lower indicating a more homogeneous deformation mode. Figure 2 shows that both NGs undergo plastic deformation mediated by multiple shear bands uniformly distributed over the whole sample.

At this point, it is interesting to compare our results obtained for 3D periodic systems with those reported for 2D periodic nanoglasses with free surfaces by Adibi et al. [14,16]. In their simulations the presence of a surfaces clearly promotes strain localization and the formation of critical shear bands, which leads to massive stress drops in the stress–strain curve. In our data for fully periodic systems the strain is obviously more delocalized and the stress drop is less pronounced. For the smallest grain sizes <5 nm, however, our data and those of Adibi et al. [14,16] agree, both showing plastic stable flow up to a large strain. This can be explained by the high fraction of interfaces, which dominate over surface effects. In a NG with a grain size smaller than 4 nm the percentage of atoms located in the soft interfaces is higher than 70%. Basically the NG structure resemble the one of a soft BMG. Similar, a transition in the deformation mechanism of a BMG form a localized deformation in shear bands to a homogeneous plastic flow was observed when increasing the casting cooling rate [26]. Here the softening of the glassy structure is due to the faster quenching, while in our case the NGs with very small grains get softer due to the high fraction of interfaces.

### Chemical composition effects

Next we test if the results on the NG mechanical properties are transferable to other Cu–Zr alloys. It is known that the short-to-medium-range structural order varies with alloy composition [27], and is assumed to play a major role in controlling the macroscopic properties of metallic glasses, particularly the plastic deformation [22,28].

**Figure 4:**
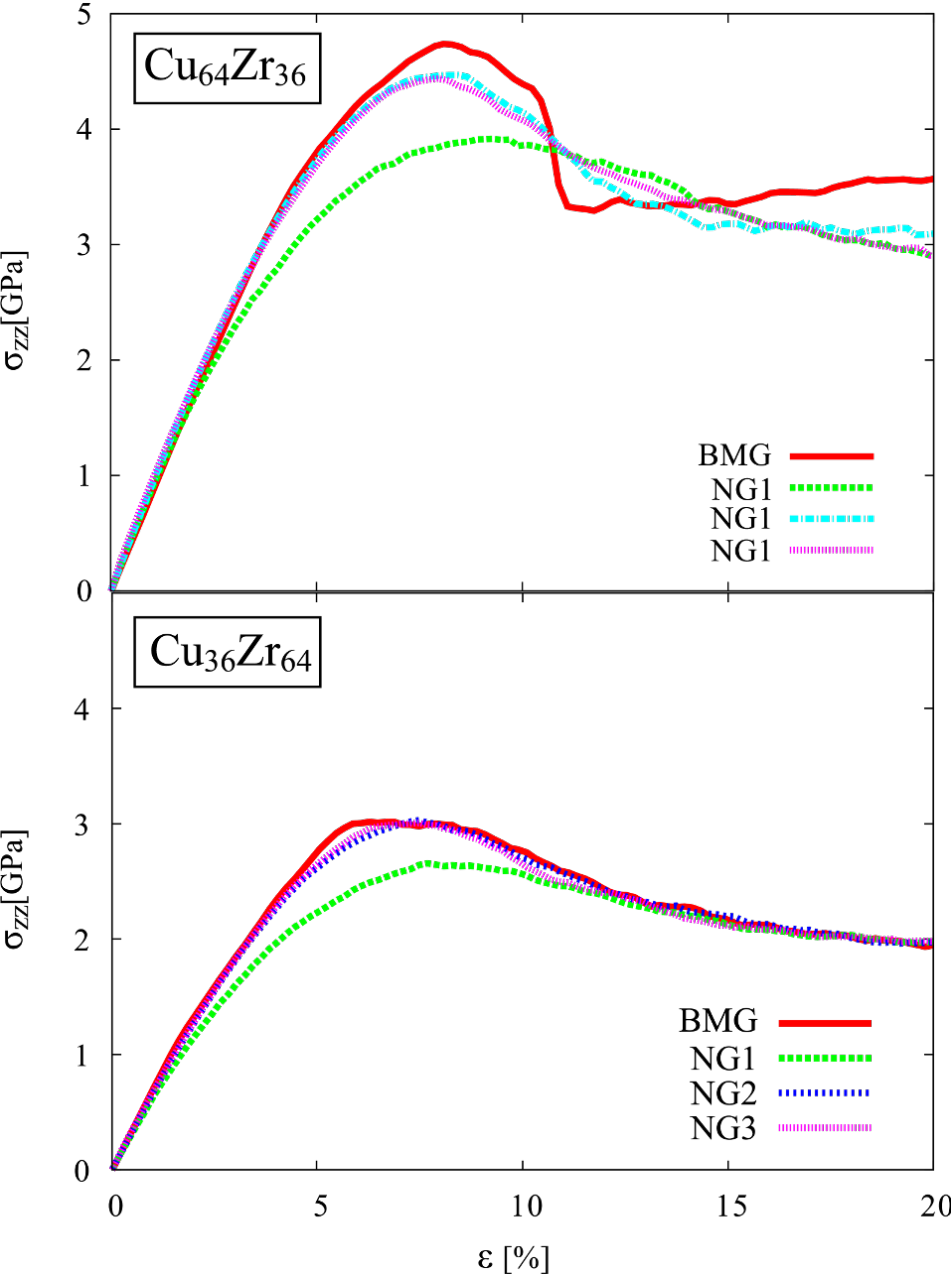
Tensile stress–strain curves for Cu_64_Zr_36_ and Cu_36_Zr_64_ BMGs and as-prepared (NG1), annealed (NG2), annealed and pre-deformed (NG3) NGs with the same chemical composition and a grain size of 10 nm, at a constant strain rate of 4 · 10^7^ s^−1^.

**Figure 5:**
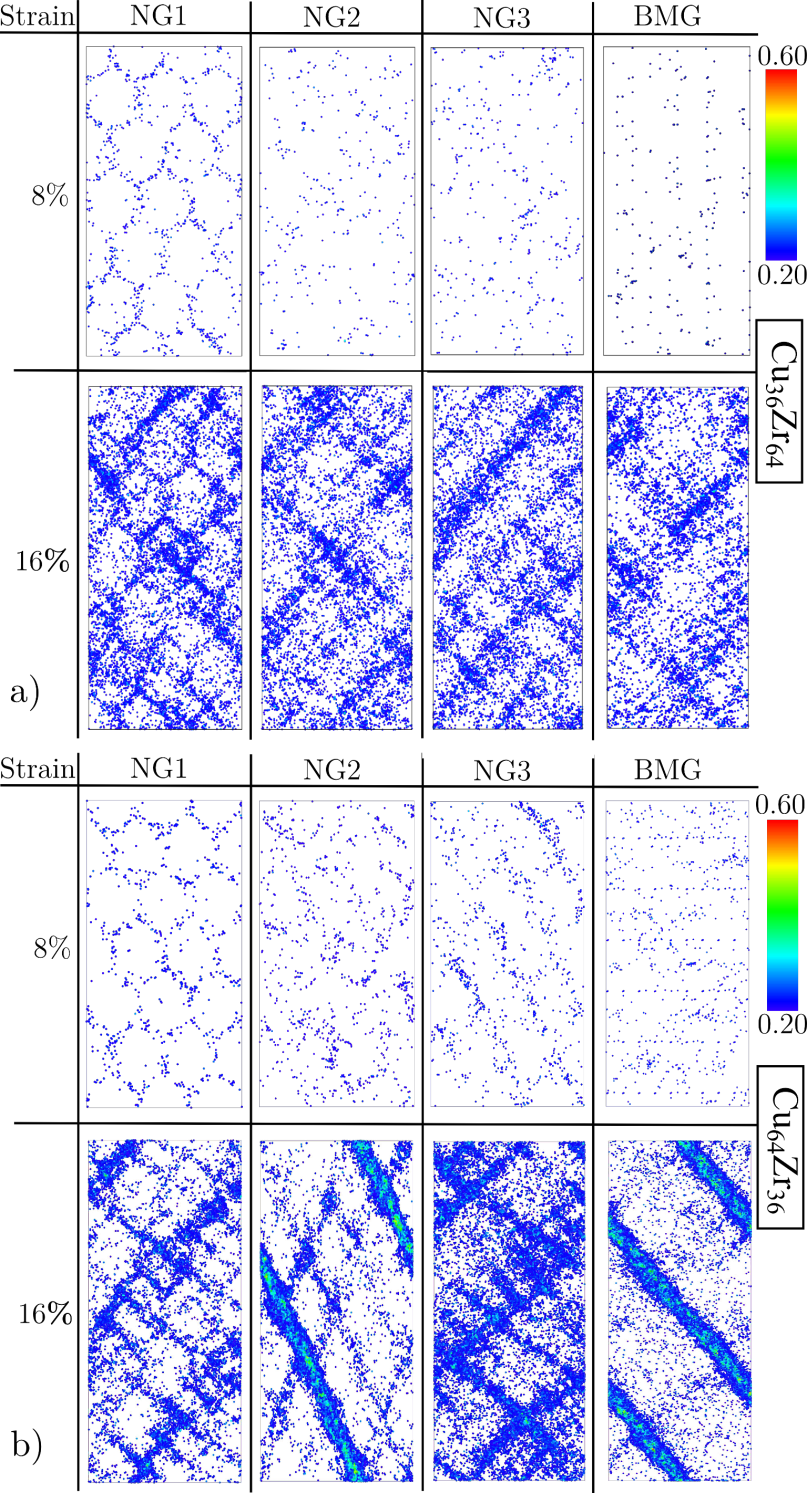
Local atomic shear strain for (a) Cu_36_Zr_64_ and (b) Cu_64_Zr_36_ BMGs and as-prepared (NG1), annealed (NG2), annealed and pre-deformed (NG3) NGs with a grain size of 10 nm.

Therefore, the influence of chemical composition on the plastic behavior of NGs was investigated by using two alloy compositions: a Zr-rich NG (Cu_36_Zr_64_) and the Cu-rich NG (Cu_64_Zr_36_) studied also in [12]. Both types of NGs have an average grain size of 10 nm. The operating deformation mechanisms in these NGs and the corresponding BMGs (≈10^6^ atoms) was analyzed under tensile deformation. In Figure 4 it can be observed that the Zr-rich BMG shows a lower yield stress compared to the yield stress of the Cu-rich BMG. In [25] it was reported that FI-clusters have a high packing density and high shear resistance and determine the plasticity of Cu–Zr glasses [21]. We found a population of Cu-centered FI in the Cu-rich alloy of about 22% compared to only 5% in the Zr-rich alloy. This can explain the corresponding variation of yield stress and plastic strain. On the other hand, it can be seen in Figure 5 that also the plastic behavior of the Cu_36_Zr_64_ BMG differs completely from the one of the Cu-rich BMG. The Cu-rich BMG exhibits localized deformation in one dominant shear band, while the Zr-rich BMG exhibits a homogeneous deformation. The difference in the short-to-medium structural order for these two alloy compositions can serve as an explanation. In the Cu_36_Zr_64_ alloy the low fraction of FI-clusters leaves space for the formation of a high fraction of Voronoi polyhedra characterized by a low packing density and low shear resistance. Hence, Zr-rich BMGs should produce more STZs and can carry more strain. On the other hand, the Cu-rich BMG contains a high fraction of FI-clusters and, therefore, a high degree of MRO formed from the interconnection of FI-units. Due to the smaller flow region within this alloy, Cu_64_Zr_36_ is unable to mediate large plastic strains. Consequently, the plastic deformation is localized in one dominant shear band. If open surface are present [14,16] both types of BMGs deform in one dominant shear band and the compositional effect is less pronounced. We repeated the deformation process of Cu_36_Zr_64_ BMG under free surface conditions and observed similar localized deformation in one dominant shear band.

**Figure 6:**
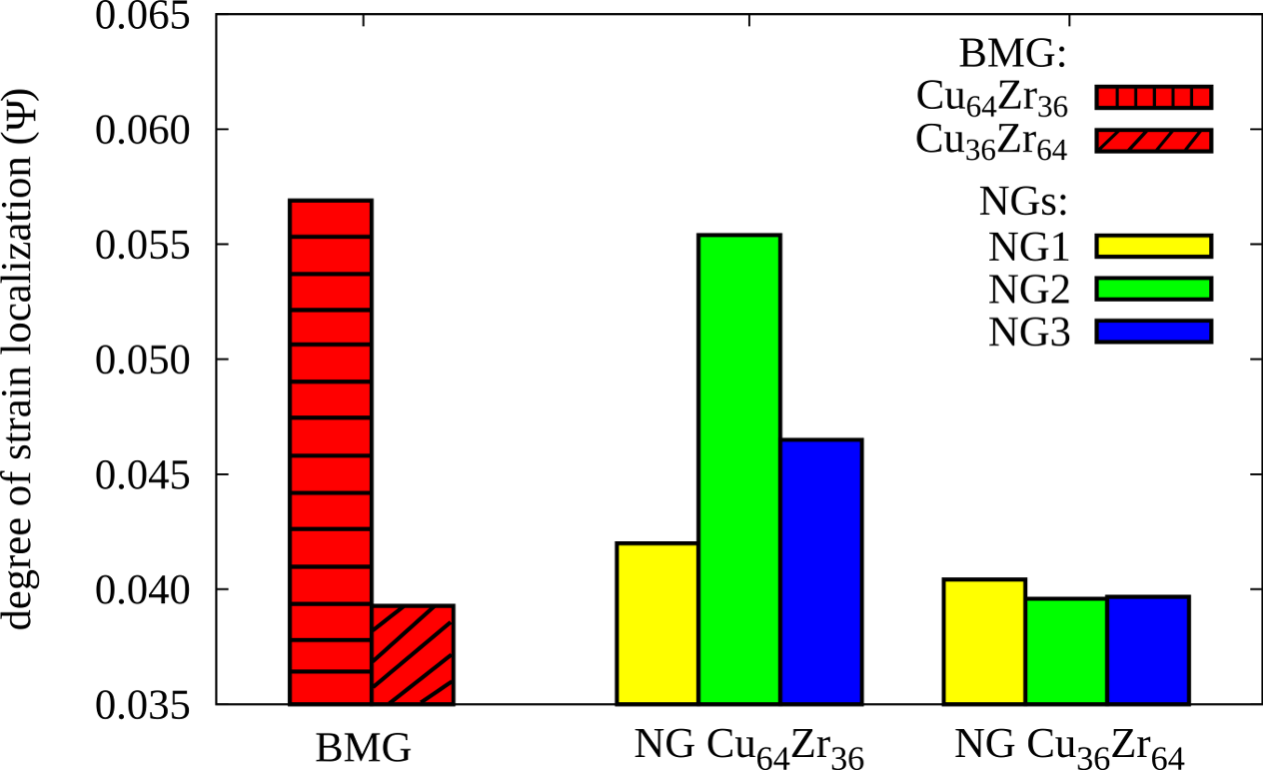
The ψ values for Cu_64_Zr_36_ and Cu_36_Zr_64_ BMGs and as-prepared (NG1), annealed (NG2), annealed and pre-deformed (NG3) NGs after plastic deformation to an overall strain of 16%.

Further, the impact of the glass–glass interface structure on the plastic behavior of Cu-rich and Zr-rich NGs is investigated in comparison. First, the stress–strain curves show a lower maximum stress for both as-prepared NGs (NG1) when compare to the BMG. In Figure 4 it can be seen that in case of as-prepared Cu_64_Zr_36_ NG the maximum stress is lower (about 4.0 GPa) compared to the BMG (about 4.7 GPa). The same trend is found for the maximum stress of NG1 Cu_36_Zr_64_ (about 2.6 GPa) compared to 3.0 GPa in case of the BMG with the same chemical composition. This can be explained by the lower activation barrier for STZs in the soft interfaces of nanoglasses [12]. This observation is supported when calculating the local atomic shear strain. Up to a strain of 8%, the STZs are only activated in the soft interface regions of both as-prepared NGs (see Figure 5, the NG1 case). Increasing the strain to 16%, embryonic shear bands are formed along the interfaces and propagate through the grain interiors. In all cases, the embryonic shear bands are blocked and no dominant shear band is formed. A more homogeneous deformation of the as-prepared Cu_64_Zr_36_ NG compared to the well localized deformation of the BMG in one dominant shear band is also supported when calculating the strain localization parameter (see Figure 6). On the other hand the Zr-rich BMG exhibits a homogeneous deformation. Therefore, in case of Cu_36_Zr_64_ NG, a more localized deformation confined in these embryonic shear bands results in a slightly higher ψ value for the NG1 compared to the BMG (see Figure 6).

The NGs studied in [14,16] have been structurally relaxed at a low temperature of 50 K. Due to the limited atoms mobilities, at this low temperature, interfaces still contain a frozen amount of excess volume. Therefore, in line with our previous studies [12] it is worth to investigate how thermal annealing affects the structure of glass–glass interfaces and the plastic deformation mechanisms in the Zr-rich NG in comparison to the Cu-rich NG. The NGs were annealed at 800 K (≈0.85 *T_g_* of the bulk glass) for 2 ns. After annealing, the NGs were quenched to 50 K and deformed in uniaxial tension. In case of annealed Cu-rich NG (NG2) the plastic deformation no longer takes place in a network of multiple shear bands, and one dominant shear band is formed (see Figure 5 lower panel). Nevertheless, as it was already shown in [12] the plastic deformation of the NG2 deviates from what has been observed for the BMG. In contrast, the deformation mechanisms of the annealed Zr-rich NG (NG2) observed at a strain of 8% and 16%, respectively, are similar to the ones in the BMG, as can be seen in Figure 5 upper panel. The anomalous plastic behavior observed for the annealed Zr-rich NG can be attributed to the structure of the interfaces for this alloy composition.

**Figure 7:**
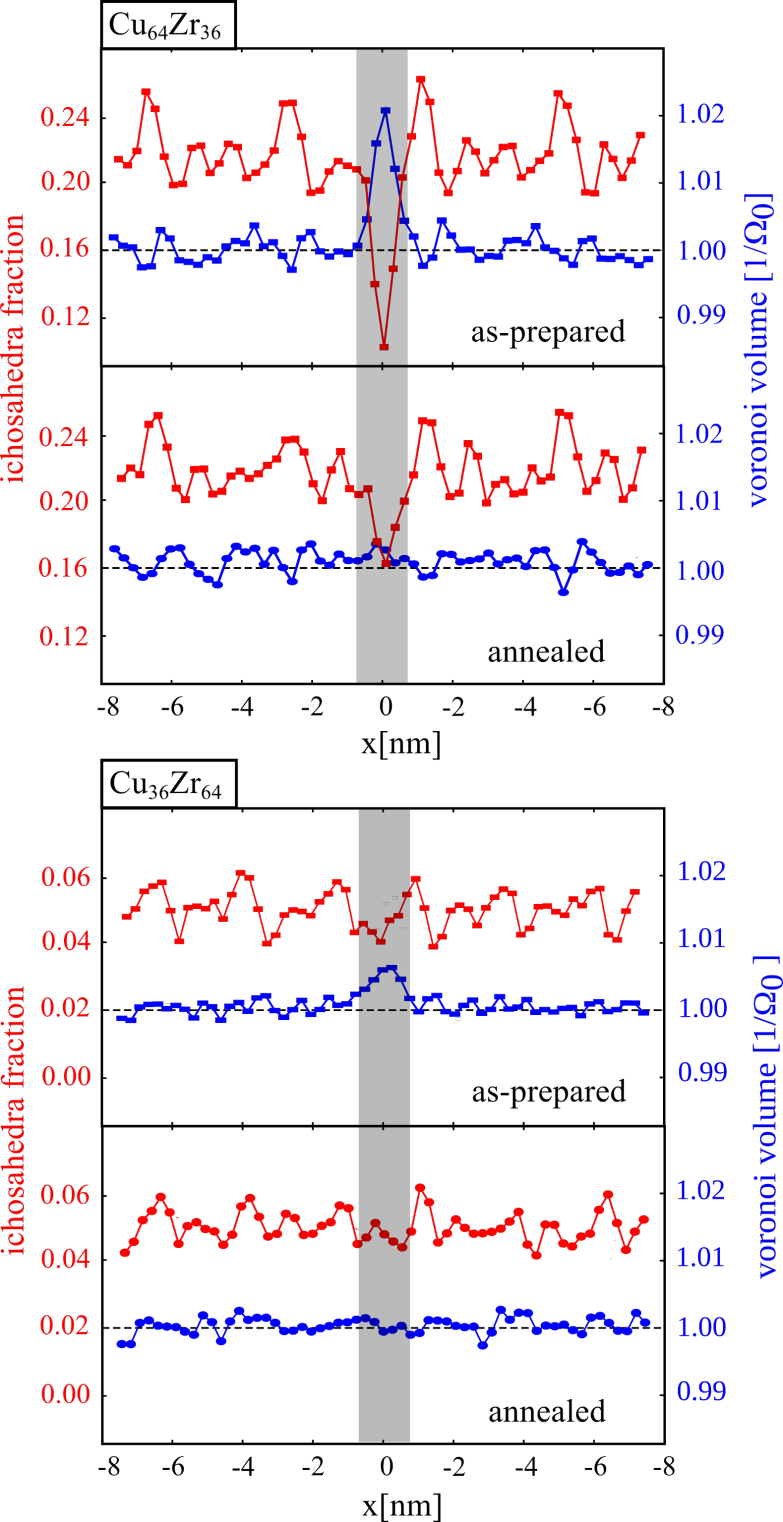
The fraction of Cu-centered full icosahedra and Voronoi volume in the Cu_64_Zr_36_ and Cu_36_Zr_64_ metallic glasses with a planar glass–glass interface.

In order to understand how the alloy composition affects the interface structure, we constructed a simple model of a planar glass–glass interface in a Cu-rich and Zr-rich glass, respectively. The interfaces were prepared by joining two planar relaxed glass surfaces. The planar interface is located at *x* = 0 parallel to the *yz*-plane. The geometry and the total number of atoms is about the same for both alloy compositions (16 × 7 × 12 nm^3^, ≈95,000 atoms). In the case of Cu_64_Zr_36_ the planar interface of about 1 nm width shows an excess free volume and a defective FI SRO. In Figure 7, upper, it can be seen that in the planar interface the FI-density is decreased by 70% from the bulk value. The fraction of Cu-centered FIs is as low as 10% in the interface compared to average FI value of the bulk glass of about 22% (with respect to the number of Cu atoms in the system). From the defective FIs SRO results approximately 1–2% excess free volume in the interface. In Figure 7, upper, the free volume through the system is plotted together with the Cu-centered FI cluster. When the Cu-rich glass with one planar interface is annealed at a temperature of 800 K for 2 ns, the fraction of FIs in the interface increases to 16% from the initial value of 10% leaving a difference between bulk glass and interface of about 2% [11]. At the same time, the excess free volume observed initially in the interfaces delocalizes completely under annealing. However, after annealing the planar interfaces in Cu-rich alloys are still characterized by a defective SRO. This explains why glass–glass interfaces in the annealed Cu_64_Zr_36_ nanoglass still have an impact on plastic deformation, as can be seen in Figure 5 upper panel.

On the other hand, in case of the Cu_36_Zr_64_ alloy the FI density is constant in the whole sample, and we do not see a deviation at the position of the interface (see Figure 7). We found that the population of Cu-centered FIs in case of the Zr-rich glass is only 5%. This value is similar to the one found in other studies [17,22]. Surprisingly, in Figure 7 it can be seen that the density in the interface is decreased although the FI density is not affected. However, the fraction of about 0.7% excess free volume detected for the interface in Zr-rich glass is much lower compared to the case of Cu-rich metallic glass (≈2%). Similar results have been found by Ritter et al. in the case of shear bands in Cu_36_Zr_64_ glass [17]. Here, the increase in the free volume in the shear band was not related to the decrease of densely packed FI cluster inside the shear bands as found for the case of Cu-rich metallic glass. In [17] it has been shown that the volume expansion inside the shear band is related to the creation of new FI clusters which show a lower packing density. The creation of these new less densely packed FI clusters fully compensate the destruction of the original icosahedra SRO. In the case of a planar interface in Zr-rich glass the increase in the free volume is related also to the destruction and formation of FI clusters. This time, the FI clusters are damaged when cutting the planar surfaces. When the interface is prepared by joining the resulting glassy surfaces a new set of FI clusters form. These new FIs show a lower packing density than the FIs in the bulk glass which leads to an increased volume inside the interface. Although the excess free volume of the planar interface in the Zr-rich glass is below 1%, it can be seen in Figure 5 lower panel that also the plastic behavior of Cu_36_Zr_64_ NG deviates from the homogeneous BMG.

Finally, we also examined, the structure of the planar interface in the Zr-rich metallic glass after thermal annealing. The system with one planar interface is annealed following the same procedure as in the case of Cu-rich glass. Also, in this case the excess free volume of the glass–glass interface in Zr-rich glass delocalizes, completely. Therefore, the planar interface in the annealed Zr-rich glass has the same FI fraction and atomic density as the bulk glass. This observation explains the similarity between the plastic behavior of the annealed Zr-rich NG and the homogeneous BMG. Without interfaces, the plasticity of the annealed NG could not be tuned by pre-deforming to strain levels below the yield stress. It can be seen in Figure 5 that even at a strain of 16% the plastic behavior of the annealed and pre-deformed NG (NG3) is almost similar to the one of the annealed NG and BMG. This observation is supported when analyzing the stress–strain curves for NG2 and NG3 in comparison the BMG. In Figure 4, it can be seen that the curves of these three structures are very similar. In addition, the degree of strain localization parameters ψ for NG2, NG3, and BMG have almost same values (see Figure 6).

## Conclusion

In the present study we investigated the influence of grain size and composition on the deformation behavior of Cu–-Zr NGs. We find that as-prepared NGs show a homogeneous plastic deformation in a pattern of multiple shear bands. For the case of Cu_64_Zr_36_ a transition from a more homogeneous to inhomogeneous plastic deformation can be observed, when the grain size is increased from 4 nm to 16 nm. Even for the largest grain size the deformation behavior of the NG differs from the well localized plastic deformation observed in the BMG. However, after a thermal annealing step, the excess free volume is equilibrating and only topological disorder can still be found in the glass–glass interfaces, which is having a minor influence on the formation of shear transformation zones. In the Zr-rich system (Cu_36_Zr_64_), in contrast, there is no appreciable difference in the topological order of the glassy matrix and the glass–glass interfaces. Thus, only the as prepared state, where some excess volume is localized in the glass–glass interfaces, shows a slightly enhanced shear localization as compared to the bulk glass.

In summary, our results show that the mechanical properties of a nanoglass can be tuned by varying the initial grain sizes, if the glass–glass interfaces have an excess free volume and are distinct in their topology from the homogeneous bulk phase.
